# Participation in research bronchoscopy: a literature review

**DOI:** 10.3402/ecrj.v3.29511

**Published:** 2016-02-03

**Authors:** Einar Marius Hjellestad Martinsen, Elise Orvedal Leiten, Per Sigvald Bakke, Tomas Mikal Lind Eagan, Rune Grønseth

**Affiliations:** 1 Department of Clinical Science, University of Bergen, Bergen, Norway; 2 Department of Thoracic Medicine, Haukeland University Hospital, Bergen, Norway

**Keywords:** COPD, clinical research, research subjects, human volunteers, motivation, refusal to participate

## Abstract

Bronchoscopy is the preferred method for collecting biological samples from the lower airways of subjects in clinical research. However, ensuring participation in clinical research can be challenging when the research includes an invasive procedure. For this report we reviewed the literature to look for information on participation in research bronchoscopy studies to better design our own study, the Bergen COPD Microbiome study (MicroCOPD). We performed a systematic literature search on participation in research bronchoscopy studies in February 2014 using the search engines of PubMed and EMBASE. The literature search resulted in seven relevant papers. Motivation was an end point in six of the seven papers, but reasons for declining participation and recruitment strategies also seemed important. Human subjects participate in research bronchoscopy studies for personal benefit and altruistic reasons. Inconvenience associated with research, in addition to fear of procedures, is considered a barrier. Radio, especially news stations, generated the most inquiries for a clinical study involving bronchoscopy. There is a lack of information on participation in research bronchoscopy studies in the literature. A bronchoscopy study has been initiated at Haukeland University Hospital, Bergen, Norway, to examine the role of the microbiome in COPD, and participation will be explored as a substudy.

Chronic obstructive pulmonary disease (COPD) will be the third leading cause of death in 2030, according to estimates by the World Health Organization (1). The mechanisms explaining why only a limited fraction of individuals exposed to tobacco and other air pollutants develop COPD remain unknown. Recent advances in the field of metagenomics have indicated that airway microbiota might differ between subjects with and without COPD (2). To sample the airway microbiota, it is necessary to have a feasible method, yet with minimal contamination. Although induced sputum is a possibility (3), this method is prone to contamination from the oral microbiota. Furthermore, the accuracy in predicting which segments of the airways are being sampled is uncertain.

Bronchoscopy is a safe procedure with a low complication rate (4) and is the ideal procedure both to ensure minimal contamination as well as to enable mapping of different areas of the airways. However, a semi-invasive procedure such as bronchoscopy can be associated with discomfort. Together with pre-procedural anxiety, this discomfort might lower participation in studies that sample the airways by bronchoscopy. Previous studies on the airway microbiota in asthma and COPD patients with bronchoscopic sampling had low numbers of participants (2, 5, 6). There is a need for studies with more statistical strength to secure reliable and reproducible data. However, large-scale bronchoscopy studies would require attention to logistic challenges, including recruitment and participation.

More information on response rates and participation motives could lead to better-targeted recruitment for clinical studies. Furthermore, by revealing common pre-procedural concerns and anxieties, it might be possible to also improve patient information and compliance in regular clinical practice. The aim of the current report was to perform a systematic review of the current literature on participation motives, response rates, and recruitment strategies in research bronchoscopy studies with an emphasis on studies including COPD patients.

## Methods

### Search strategy

Two separate literature searches were performed using the PubMed search engine of the US National Library of Medicine (7) and the Excerpta Medica Database (EMBASE) provided by the medical publisher Elsevier (8).

PubMed papers are indexed by keywords called *medical subject headings* (MeSH) (9). Due to the hierarchical organization, generalized MeSH terms include papers classified by specific MeSH term. We identified MeSH terms from the indexed papers in initial searches, supplied by qualified suggestions from a collegial brainstorming session using a modification of a population–intervention–comparison–outcome (PICO) scheme (10). Most search terms were included as both MeSH terms and text words to increase search sensitivity. The columns were combined with ‘OR’, and rows were combined with ‘AND’.

EMBASE has similar functions as PubMed, though MeSH terms are replaced by Emtree terms. We used the same modified PICO scheme (Table 1) for the EMBASE search. MeSH terms were replaced by explosion search, and text words were replaced by multipurpose (mp) terms.

**Table 1:** Modified PICO scheme used for a literature review on participation in research bronchoscopy studies

P1	P2	I	O
COPD (MeSH)	Patients (MeSH)	Bronchoscopy (MeSH)	Patient participation (MeSH)
COPD (tw)	Patients (tw)	Bronchoscopy (tw)	Participation (tw)
Chronic obstructive pulmonary disease (tw)	Participants (tw)		Response (tw)
	Human volunteers (MeSH)		Non-response (tw)
	Volunteers (tw)		Attitude (MeSH)
	Study (MeSH major topic)		Attitude (tw)
	Trial (MeSH major topic)		Motivation (MeSH)
	Research subjects (MeSH)		Motivation (tw)
	Research subjects/psychology (MeSH)		Refusal to participate (MeSH)
	Clinical research (MeSH)		Refusal to participate (tw)
			Informed consent (MeSH)
			Participation rate, patient (MeSH)
			Participation rate (tw)
			Patient selection (MeSH)
			Patient selection (tw)
			Advertising as topic (MeSH)
			Advertising (tw)
			Risk assessment (MeSH)
			Risk assessment (tw)
			Altruism (MeSH)
			Altruism (tw)
			Perception (tw)

The P1 column was excluded in the final search due to a paucity of results. P, population; I, intervention; C, comparison; O, outcomes; MeSH, medical subject headings; tw, text words

Titles and abstracts were sifted and classified by prespecified exclusion and inclusion criteria (Table 2). Only papers concerning recruitment to studies including bronchoscopy were included. Reports of motives or perceived benefits of participation in studies with respiratory invasive procedures, reasons for non-response, recruitment sources, and response rates in studies involving respiratory invasive procedures were included. Papers not written in English or a Scandinavian language were excluded, together with non-human studies, case studies, and secondary publications, including literature reviews, reports, comments, letters, guidelines, newspaper articles, books, or book chapters. Studies that did not have participation as a main objective or as a study end point were also excluded. Similar criteria were used in evaluating retrieved papers found from both the PubMed and EMBASE searches.

**Table 2:** Number of retrieved papers for a literature search in the databases PubMed and EMBASE on participation in research bronchoscopy studies according to classification criteria

Classification criteria	PubMed	EMBASE
**Total number retrieved**	**989**	**987**
Non-English/non-Scandinavian language	102	None
Case studies/series	82	427
Secondary publications	116	108
Non-human studies	7	None
Participation not a major topic	674	443
**Papers included in review**	**8**	**9**
Papers common to PubMed and EMBASE searches	7	

Secondary publications included reviews, expert panels, letters, guidelines, and so on. The EMBASE search excluded studies in languages other than English and Scandinavian languages, as well as non-human studies

## Results

Results from the two literature searches were classified as shown in Table 2. The majority of papers, 1,117, were excluded due to their lack of participation as a main objective or study end point. The PubMed search yielded eight relevant papers, and the EMBASE search yielded nine relevant papers. Seven out of nine articles from the EMBASE search were also found in the PubMed search. Thus, 10 individual papers were included for in-depth review. Three of these 10 papers did not report participation and were excluded.

Table 3 provides an in-depth overview of the final seven included papers (11–17). Four of the papers were published in the last five years, and six of the studies were conducted in Europe. Six papers focused on motives or perceived benefits of participation for studies involving research bronchoscopies (11, 12, 14–17). One of these also reported reasons to decline participation (16), and one studied predictors for the decision to consent to a second bronchoscopy (14). Further, one study evaluated the recruitment process in a lung cancer chemoprevention study that included a research bronchoscopy (13). Research bronchoscopies were carried out in all the studies (Table 3).

**Table 3:** Overview of the included papers after a literature search on participation in research bronchoscopy studies

Author, year of publication, country (reference)	Study objective	Main outcomes	*N*	Population	Inclusion criteria	Design	Purpose of bronchoscopy	Bronchoscopy procedure	Relevant findings
Lipman, 1998, United Kingdom and United States (14)	Examine acceptability of research bronchoscopy in asymptomatic HIV subjects	Pre- and post-bronchoscopic participation motives, willingness to undergo second procedure. Response rates	Responders: 75 Non-responders: 23	HIV-infected individuals, 18–60 years	No acute/chronic respiratory disease, IV drug use or antiretroviral therapy	Prospective semistructured, self-completed questionnaires	Harvest cell populations from the lung	Bronchoscopy with BAL	Response rate: 70% Participation motives: personal benefits, altruism Non-responders younger 79% would accept second research bronchoscopy
Mtunthama, 2008, Malawi and United Kingdom (15)	Examine adequacy of study information, motives for participation, and complication rate in a research bronchoscopy study	Participation motives, perceived sufficiency of study information, self-reported adverse events	100	Malawian volunteers	None given	Prospective. Interviews with both open and closed questions	Determine factors underlying susceptibility to respiratory infection among adults	Bronchoscopy with BAL	Response rate: not given Participation motives: personal benefit, study enthusiasm All agreed to a second bronchoscopy. 94% considered pre-information adequate and useful
Kerrison, 2008, United Kingdom (12)	Examine patients’ experience of clinical research	Participation motives, importance of referral and consent procedures, sufficiency of study information	18 in bronchoscopy study	Six clinical studies	None given	Retrospective qualitative study. Telephone interviews, questionnaires or focus groups	Identify and sample suspicious lesions	Fluorescence bronchoscopy and optical biopsy	Response rate: 64% in bronchoscopy substudy Participation benefits: extra care, increased surveillance, see an expert and help researchers and others Most encountered studies through the caregiving hospital
Kye, 2009, USA (13)	Examine various recruitment strategies	Response rates and cost calculations	137	Healthy ex-smokers, ≥30 pky	No contraindications for bronchoscopy or celecoxib, no use of certain medications or no cancer	Prospective study. Telephone interviews	Examination and sampling	White light and fluorescence bronchoscopy with BAL and bronchial biopsies	Response rate: 3.1% Radio advertisements generated most inquiries, followed by Internet, print media, posted flyers, and mass mailing
Schook, 2010, Netherlands (17)	Examine whether participation in a smoking cessation trial could influence smoking cessation	Smoking cessation rate and influence of participation on cessation	146	Healthy current or former smokers, ≥20 pky	No serious comorbid disease, FEV1 below 1000 mL or use of systemic or inhaled corticosteroids in previous year	Retrospective study. Questionnaire-based telephone interviews	Examination	Autofluorescence or white light bronchoscopy with biopsies	Response rate: 73% Participation motives: personal benefit
Patel, 2012, United Kingdom (16)	Examine methods and participation in a lung cancer screening study	Participation motives, declining reasons, and views on screening method	60	Current or ex-smokers, ≥20 pky and or ≥20 years, with mild to moderate COPD	No serious comorbid disease, life expectancy ≥5 years	Prospective qualitative study. Semi-structured interviews	Examination	Fluorescence bronchoscopy with biopsies	Response rate: not given Participation motives: personal benefit, altruism Participation barriers: fear, bad experiences, and travel
Chudleigh, 2013, United Kingdom (11)	Examine recruitment and retention of CF infants and healthy controls, as well as parental attitudes to participation	Participation motives, benefits, and disadvantages. Response rates	Cases: 85 Volunteers: 56	Infants with and without CF, including parents	CF: no contraindicated disorders, no preterms. Healthy controls: no medical and/or social contraindications, ≥2500 g	Prospective longitudinal, observational study. Self-completed questionnaires	Examination	Bronchoscopy with BAL	Response rate: 69% (CF), 21% (healthy controls) Participation motives: personal benefit, altruism

BAL, bronchoalveolar lavage; COPD, chronic obstructive pulmonary disease; CF, cystic fibrosis; FEV1, Forced expiratory volume; pky, pack-years.

Five of the reviewed studies were prospective (11, 13–16). The largest included 146 participants in a smoking cessation trial (17), whereas the smallest examined 18 subjects (12). Three studies were limited to current or ex-smokers as study subjects (13, 16, 17), and none of these studies compared results with a healthy control population. Only one study included COPD patients (16), but the study emphasized lung cancer. Thus, none of the studies published results generalizable to a COPD population.

The most frequently used method of obtaining information was interviews (12, 13, 15–17), which were conducted by telephone in three of the studies (12, 13, 17). The remaining two studies made use of self-completed questionnaires for data collection (11, 14). Statistical methods were not reported in three of the included studies (11–13). The effects of demographic variables on participation were examined in five of the papers (13–17).

### Motivation and benefits of study participation

We identified four main groups of motives for participation in bronchoscopy studies – *personal benefit* (11, 12, 14–17), *altruism* (11, 14, 16), *perceived importance of research* (11, 12), and *obedience to the authority of the researchers* (14).

*Personal benefit* was found as a participation motive in all six papers that examined participation motives (11, 12, 14–17). The benefits appeared to take various forms, but were mainly defined as interest in their own health (11, 14, 17), getting a proper health assessment (15, 16), or treatment or surveillance of their health (12, 15). In the study of parents of children with cystic fibrosis (CF), personal benefit was more important when they accepted participation on behalf of their children compared to parents of healthy control infants (98 vs 25%) (11). In the Malawian study, new volunteers expected participation to be of benefit to them, perceived as health assessment and prompt treatment (15). In the study on an HIV-infected population in the United Kingdom, two-thirds of participants stated personal benefit as important, but only 51% gave their own health as their main motive for participation (14).

In the latter study (14), *altruism* was considered the main reason for participation by HIV-infected patients. The same was true for parents of healthy controls in the CF case–control study (11). In the study by Patel et al., four of seven elderly participants (age >70) gave altruistic reasons for participation. However, this motive was often accompanied by self-interest. No participants stated altruism as the only motive for participation (16).

Participation as a result of *physician's authority* was rare, but could be seen in the study by Lipman et al. (14), which found that participants were motivated by being asked by a physician or that the physician seemed to want them to participate. Kerrison et al. (12) found a somewhat similar motive when participants described their participation as an *important investment in scientific progress*. This motive was also mentioned by 32% of the parents in the CF case–control study (11).

### Response rates

*Response rate*, defined as number of enrolled divided by approached or prescreened individuals, was a main objective in three of the reviewed papers (11, 13, 14), whereas response rates could be found or derived from two additional papers (12, 17). Response rates varied from 3 to 73% (11, 17) and seemed to be higher in studies involving individuals that were affected by the index disease (11, 14). Chudleigh et al. showed that recruitment of healthy controls was feasible, but more challenging than recruiting CF patients (11). Only Lipman et al. looked into predictors for participation and found that participants were significantly older (14).

### Refusal

Reasons for declining primary participation or a second bronchoscopy was reported in three articles (13, 14, 16), and disadvantages of participation were examined in one study (11). All studies exploring refusal to participate listed a negative view on bronchoscopy as a main reason for non-response (13, 14, 16), and the severity and duration of previous experienced post-bronchoscopy symptoms was the most common reason to refuse a second research bronchoscopy in an HIV population (14). Patients that refused or were unsure (21% of participants who already had undergone a bronchoscopy) had more clinically advanced HIV infection. However, all of the participants did agree to a second bronchoscopy if medically indicated (14). Patel et al. identified barriers to participation as disadvantages of involvement exemplified by travel inconvenience, bad experiences, and negative perceptions of bronchoscopy (16), whereas Chudleigh reported anxiety and perceived risk for complications as negative aspects of participation (11).

### Recruitment strategies

The study by Kye et al. was the only one reporting the efficacy of various recruitment strategies, and in a US lung cancer screening trial they found that radio advertisement was the most effective, especially information on the news stations, followed by Internet posting, print media, posted and racked flyers, and mass mailings (13).

## Discussion

We have shown that the literature on participation in research bronchoscopy studies is somewhat limited. Nevertheless, investigators planning new studies might benefit from some inferences. First, it seems that both control subjects and younger individuals have lower response rates (11, 14). The highest response rates were seen in a study involving current or former smokers (17) and in a study of an HIV population (14). Conversely, healthy subjects in a chemoprevention study had the lowest participation rate (3.1%) (13), possibly suggesting that more advanced diseases result in higher participation fractions. However, the latter study also pointed out the challenge of recruiting healthy subjects, as these require strict entry criteria and minimal comorbidities, which may also have contributed to their very low response rate. An earlier review conducted on participation in COPD studies without an invasive procedure also examined response rates (18). Sohanpal et al. found that study participation rates were higher than expected, and 81% of the studies included had a study participation rate above 50%. This finding conforms to the current report. The average participation rate was 77.8% in Sohanpal et al.'s review, whereas our review had an average of 55.8%, possibly suggesting higher participation rates in studies without invasive procedures. However, our material is limited and caution needs to be taken when comparing these results. Second, it seems that the main motivation for participation lies somewhere in a balance between perceived personal health benefit and altruism. This warrants some caution from investigators in not portraying participation as a substitution for otherwise inadequate healthcare access and some modesty in what results might be expected from the study. In particular, professionals should be aware that perceived authority results in undue pressure on invitees (14). Third, the main reason for non-response or declination of participation was fear related to the invasive procedure. It would be logical to assume that both content and deliverance of study information influences participation, but no study examined these factors in detail. Fourth, participation in research bronchoscopy does not seem to negatively influence patient consent for medically indicated procedures (14), which is of key importance when deciding whether or not to include bronchoscopy in a clinical study. Finally, researchers considering media as a recruitment source should know that radio and Internet advertising seem to be the most effective sources (13).

The distinction between non-therapeutic and therapeutic studies could possibly give rise to different participation motives. In the current review, we defined three of the articles as therapeutic (13, 16, 17), defined by any perceived direct benefit to the participants involved, and four as non-therapeutic (11, 12, 14, 15). Interestingly, all six papers that focused on motivation (11, 12, 14–17) reported personal benefit to be important. The perceived benefit from a non-therapeutic study could reflect a lack of understanding among the participants, thus emphasizing the importance of providing adequate and detailed information to eligible subjects.

In comparison to our area of study, participation in colorectal cancer screening trials was reviewed by Bakker et al. (19). They found that participation rates and completion of the fecal occult blood test as a screening procedure was higher when the researcher added the screening kit to the invitations. The addition was compared to invitations in which participants had to request the kit if interested or visit their general practitioner or a screening center to obtain the kit. In addition, a higher test completion rate was observed if participants received the test by post before a health check rather than being offered the test at the health check. General practitioner involvement and face-to-face invitation also resulted in higher participation. Furthermore, long travel distances from the screening facility were considered a participation barrier, consistent with results reported in the study by Patel et al. included in the current review (16).

A review by Ellis assessed patient and physician participation in randomized clinical trials in oncology (20). In line with our findings, the review confirmed that altruism, personal benefit, and scientific contribution were important motives for participants. Physician's authority was not emphasized, but patients that trusted their doctors seemed more willing to participate in clinical trials. Males, older patients, less well-educated persons or persons from lower socioeconomic backgrounds were also more inclined to participate. Among the reasons for non-response the authors emphasized fear of randomization, suggesting the process to be unfair, and loss of freedom to make their own decisions. Some individuals reported the feeling of being a guinea pig as unpleasant. Lack of information and distrust of the medical profession also appeared as barriers. Further, large difference in the treatment offered negatively influenced the decision to participate.

Our review has also revealed some limitations in the existing literature and some fields that warrant further research. The bulk of literature was on participation motives, whereas only two studies presented information on non-responders. Only one of these two presented a response rate, but this study had fewer than 100 individuals in their final analysis, and only 52% of the participants had actually undergone a bronchoscopy at the time of analysis (14).

We have summarized the existing literature on recruitment strategies, response rates, and participation motives in studies including bronchoscopy as a part of their design. In particular we set out to identify studies including COPD patients, but found there was very little data on this patient group. Inclusion for an observational bronchoscopy study was completed in June 2015 at Haukeland University Hospital, Bergen, Norway (MicroCOPD). The aim of the MicroCOPD study is to shed light on the role of the microbiome in COPD (21). Participants underwent a bronchoscopy with collection of protected specimen brushes, small-volume lavage, bronchoalveolar lavage, and bronchial biopsies. The respiratory microbiome in subjects with and without COPD will be investigated relative to disease progression and development and are expected to provide insight in a new and promising research field. Participation will be examined as a substudy. Motives for participation will be asked as an open question before the bronchoscopy. Response rates will be estimated, and predictors for participation are targeted to be revealed. It is anticipated that these results will contribute to later research on COPD and facilitate the conduction of other bronchoscopy studies.

## Conclusions

A literature search performed between December 2013 and February 2014 exploring participation in clinical studies involving research bronchoscopies yielded seven relevant articles. Conducting bronchoscopy studies involves difficulties in recruiting control subjects and younger individuals, as well as the invasive nature of the procedure. Responders seem driven by a combination of personal health benefit and altruistic motives. However, we found no solid evidence on recruitment for COPD studies, and the characterization of non-responders had major limitations. Thus, further research on participation in bronchoscopy studies is warranted.
